# Local structures around the substituted elements in mixed layered oxides

**DOI:** 10.1038/srep43791

**Published:** 2017-03-02

**Authors:** Shota Akama, Wataru Kobayashi, Kaoru Amaha, Hideharu Niwa, Hiroaki Nitani, Yutaka Moritomo

**Affiliations:** 1 Graduate School of Pure and Applied Science, University of Tsukuba, Tsukuba 305-8571, Japan; 2 Faculty of Pure and Applied Science, University of Tsukuba, Tsukuba 305-8571, Japan; 3 Tsukuba Research Center for Interdisciplinary Materials Sciences (TIMS), University of Tsukuba, Tsukuba 305-8571, Japan; 4 Center for Integrated Research in Fundamental Science and Engineering (CiRfSE), University Tsukuba, Tsukuba 305-8571, Japan; 5 Institute of Materials Science, High Energy Accelerator Research Organization (KEK), Tsukuba 305-0801, Japan

## Abstract

The chemical substitution of a transition metal (*M*) is an effective method to improve the functionality of a material, such as its electrochemical, magnetic, and dielectric properties. The substitution, however, causes local lattice distortion because the difference in the ionic radius (*r*) modifies the local interatomic distances. Here, we systematically investigated the local structures in the pure (*x* = 0.0) and mixed (*x* = 0.05 or 0.1) layered oxides, Na(*M*_1−*x*_*M*′_*x*_)O_2_ (*M* and *M*′ are the majority and minority transition metals, respectively), by means of extended X-ray absorption fine structure (EXAFS) analysis. We found that the local interatomic distance (*d*_*M*-O_) around the minority element approaches that around the majority element to reduces the local lattice distortion. We further found that the valence of the minority Mn changes so that its ionic radius approaches that of the majority *M*.

The chemical substitution of a transition metal (*M*) is an effective method to improve the functionality of a material, such as its electrochemical^1^,^2^,^3^,^4^,^5^,^6^ magnetic^7^,^8^,^9^ and dielectric^10^ properties. For example, NaFe_0.5_Co_0.5_O_2_^1^ with an O3-type layered structure shows a high discharge capacity of 160 mAh/g and good cyclability, which is much higher than those of the parent O3-NaFeO_2_ and O3-NaCoO_2_. In addition, Na_0.67_Fe_0.5_Mn_0.5_O_2_^2^ with a P2-type layered structure shows a high discharge capacity of 190 mAh/g, which is much higher than that of the parent P2-Na_0.67_MnO_2_. To thoroughly comprehend the effects of a partial substitution on the material properties, we first have to know the local structures around the respective *M*s, e.g., the interatomic distance and its valence. The extended X-ray absorption fine structure (EXAFS) and X-ray absorption near-edge structure (XANES) analyses around the K-edges are powerful techniques to determine the local structures around the respective *M*s in the mixed compounds. Let us consider the situation in which the ionic radius (*r*) of the minority (substituted) *M* is larger than that of the majority (host) *M*. If the valence state of the minority *M* is extremely stable, the interatomic distance between the minority *M* and a neighboring atom is the same as in the parent compound that consists of the minority *M*. In this case, the resultant local lattice distortion around the minority *M* increases the Gibbs free energy. Hereafter, we will call this change in energy “distortion energy”. In contrast, if the distortion energy around the minority *M* is extremely high, *M* tends to take the same interatomic distance as in the majority *M*. In this case, the valence state of the minority *M* may change so that its ionic radius approaches that of the majority *M*. The actual systems are considered located between these two extreme cases. Thus, the local structure is governed by a subtle competition between the local distortion and the valences.

Among the transition metal compounds, the mixed layered oxides, Na(*M*_1−*x*_*M*′_*x*_)O_2_ (*M* and *M*′ are the majority and minority transition metals, respectively)^1^ are promising candidates for the cathode material for a sodium-ion battery^11^, (2) have large families with different combinations of transition metals, and (3) show simple crystal structure with alternating *M*O_2_ layers and Na sheets. The *M*O_2_ layer consists of edge-sharing *M*O_6_ octahedra formed by covalent bonding. In this sense, the layered oxides are suitable systems for the investigation of the local structures. The layered oxides have two crystal structures, *i.e*., the O3- and P2-types, depending on the stacking pattern of the *M*O_2_ layers and Na sheets. The oxygen sheets of the *M*O_2_ layers stack as AB|CA|BC (AB|BA) in the O3-type (P2-type) compounds, where the vertical line represents the Na sheet.

Here, we systematically investigated the interatomic distance around the respective *M*s and their valence states in the mixed layered oxides. The systematic EXAFS analyses revealed that the local interatomic distance (*d*_*M*-O_) around the minority *M* approaches that around the majority *M*. A similar effect is discernible in the interatomic distance (*d*_*M*-*M*_) between the neighboring *M*s. We further found that the valence of the minority Mn changes so that its ionic radius approaches that of the majority *M*.

## EXAFS analysis

**Figure 1:**
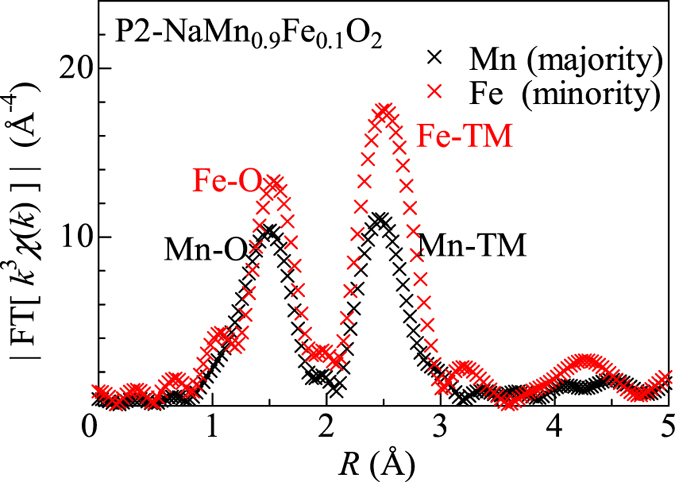
FT[*χ*(*k*)*k*^3^]−*R* plots of the majority Mn and minority Fe in P2-NaMn_0.9_Fe_0.1_O_2_. The curves were obtained by Fourier transformation of the *χ*(*k*)*k*^3^−*k* plots in the *k*-range from 2 Å^−1^ to 11.2 Å^−1^, where χ and k are the oscillatory components of the normalized absorption and angular wavenumber, respectively.

EXAFS analysis is one of the most powerful methods to determine the local interatomic distance around the respective *M*s in these mixed compounds. Figure 1 shows a prototypical Fourier transformation of the *χ*(*k*)*k*^3^ − *k* plots around the majority Mn and minority Fe in P2-NaMn_0.9_Fe_0.1_O_2_. χ and k are the oscillatory components of the normalized absorption and angular wavenumber, respectively. Both the plots show a two-peak feature, which is attributable to the paths to the first- (O) and second- (TM) nearest neighbor elements, respectively. The peak positions (1.50 and 2.47 Å) in the Mn plot are slightly smaller than those (1.55 and 2.52 Å) in the Fe plot. This indicates that the interatomic distances around the majority Mn are shorter than those around the minority Fe. To evaluate precisely the interatomic distances, we performed least-squares fitting of the FT[*χ*(*k*)*k*^3^] − *R* plots with the EXAFS equation (see Method section). In the O3 compounds, we included the contributions from the first- (O), second- (*M*), and third- (Na) nearest neighbor elements. In the P2 compounds, we included the contributions from the first- (O), third- (*M*), and fifth- (O) nearest neighbor elements. We omitted the contribution from the second- (Na2) and fourth- (Na1) nearest neighbor elements because their occupancies (*g *=* *0.21 – 0.41) are small. The interatomic distances thus obtained, *i.e*., *d*_*M*-O_ and *d*_*M*-*M*_, are listed in Table 1.

## Interatomic distance between *M* and O

**Figure 2:**
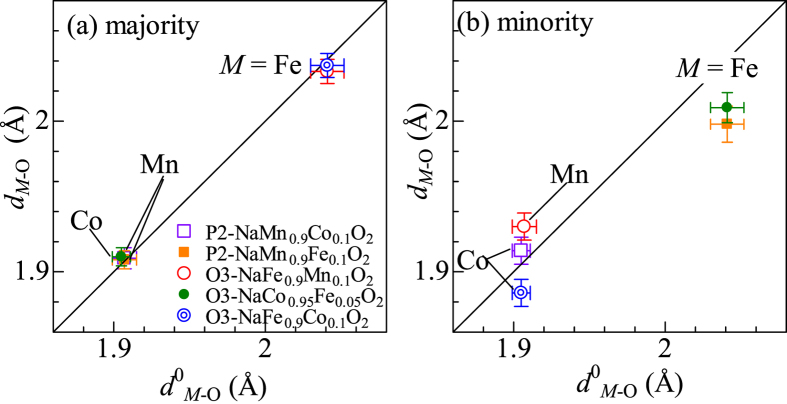
Interatomic distances (*d*_*M*-O_) between *M* and O in the mixed layered oxides around (**a**) majority and (**b**) minority *M*s. Horizontal axes are the *M*-O distances (*d*^0^_*M*-O_) in the corresponding pure layered oxides, *i.e*., O3-NaFeO_2_, O3-NaCoO_2_, and P2-NaMnO_2_.

In Fig. 2, we plotted the interatomic distances (*d*_*M*-O_) between *M* and O in the mixed layered oxides against those (*d*^0^_*M*-O_) in the corresponding pure layered oxides, *i.e*., O3-NaFeO_2_, O3-NaCoO_2_, and P2-NaMnO_2_. In the majority *M* [Fig. 2(a)], the *d*_*M*-O_ values coincide with the *d*^0^_*M*-O_ values within experimental error, indicating that the partial (<10%) substitution does not influence the local structure around the majority *M*.

In the minority *M* [Fig. 2(b)], however, considerable differences are observed between *d*_*M*-O_ and *d*^0^_*M*-O_. In the Fe-substituted compounds, *i.e*., O3-NaCo_0.95_Fe_0.05_O_2_ and P2-NaMn_0.9_Fe_0.1_O_2_, *d*_Fe-O_ (=2.009 and 1.998 Å) is shorter than *d*^0^_Fe-O_ (=2.041 Å) by 1.6–2.1%. We note that the *d*_Fe-O_ of the minority Fe is much longer than *d*^0^_Co-O_ (=1.905 Å) and *d*^0^_Mn-O_ (=1.907 Å). In short, *d*_Fe-O_ of the minority Fe approaches *d*^0^_Co-O_ and *d*^0^_Mn-O_. A similar trend is observed in the Mn-substituted compound. In O3-NaFe_0.9_Mn_0.1_O_2_, *d*_Mn-O_ (=1.930 Å) is longer than *d*^0^_Mn-O_ (=1.907 Å) by 1.2% and approaches *d*^0^_Fe-O_ (=2.041 Å). We note that the same trend is observed even if we only include the contributions from the nearest neighboring O and *M* in the EXAFS analyses (Fig. S12).

## Interatomic distance between *M* and *M*

**Figure 3:**
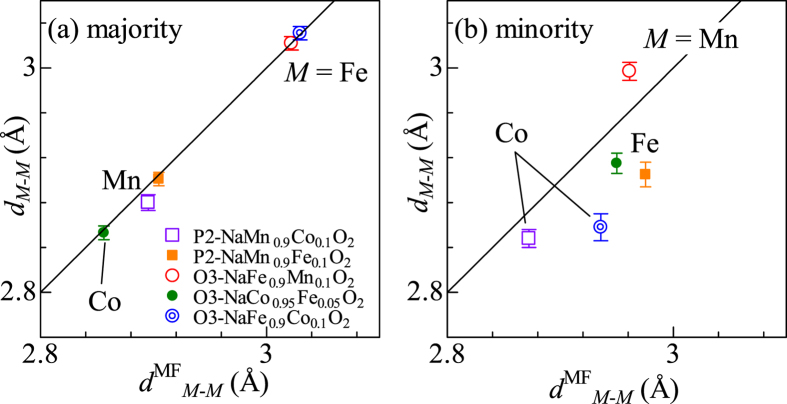
Interatomic distances (*d*_*M-M*_) between the neighboring *M*s in the mixed layered oxides around (**a**) majority and (**b**) minority *M*s. Horizontal axes are the mean-field values (*d*^MF^_*M-M*_) evaluated from the values of the pure layered oxides, *i.e*., O3-NaFeO_2_, O3-NaCoO_2_, and P2-NaMnO_2_.

In Fig. 3, we plotted the interatomic distances (d_*M*-*M*_) between the neighboring *M*s in the mixed layered oxides against the mean-field values (*d*^MF^_*M-M*_). The mean-field values are expressed, for example, in NaFe_1-y_Co_y_O_2_, as *d*^MF^_Co-*M*_ = (1 − *y*)*d*^0^_Fe-Fe_ + *yd*^0^_Fe-Co_ around Fe and *d*^MF^_M-M_ = (1 − *y*)*d*^0^_Co-Fe_ _+_ *yd*^0^_Co-Co_ around Co, where *d*^0^_Fe-Fe_ and *d*^0^_Co-Co_ are the values in O3-NaFeO_2_ and O3-NaCoO_2_, respectively. The value *d*^0^_Fe-Co_ (=*d*^0^_Co-Fe_) is defined by (*d*^0^_Fe-Fe_ + *d*^0^_Co-Co_)/2. In the majority *M* [Fig. 3(a)], *d*_*M-M*_ coincides with the mean-field values within experimental error.

In the minority *M* [Fig. 3(b)], however, considerable differences are observed between *d*_*M-M*_ and *d*^MF^_*M-M*_. In the Fe-substituted compounds, *i.e*., O3-NaCo_0.95_Fe_0.05_O_2_ and P2-NaMn_0.9_Fe_0.1_O_2_, *d*_Fe-*M*_ is shorter than the mean-field value, reflecting the compressed *d*_Fe-O_ (<*d*^0^_Fe-O_). A similar trend is observed in the Mn-substituted compound. In O3-NaFe_0.9_Mn_0.1_O_2_, *d*_Mn-*M*_ is longer than the mean-field value, reflecting the elongated d_Mn-O_ (>*d*^0^_Mn-O_). We note that the same trend is observed even if we only include the contributions from the nearest neighboring O and *M* in the EXAFS analyses (Fig. S13).

## XANES spectra

**Figure 4:**
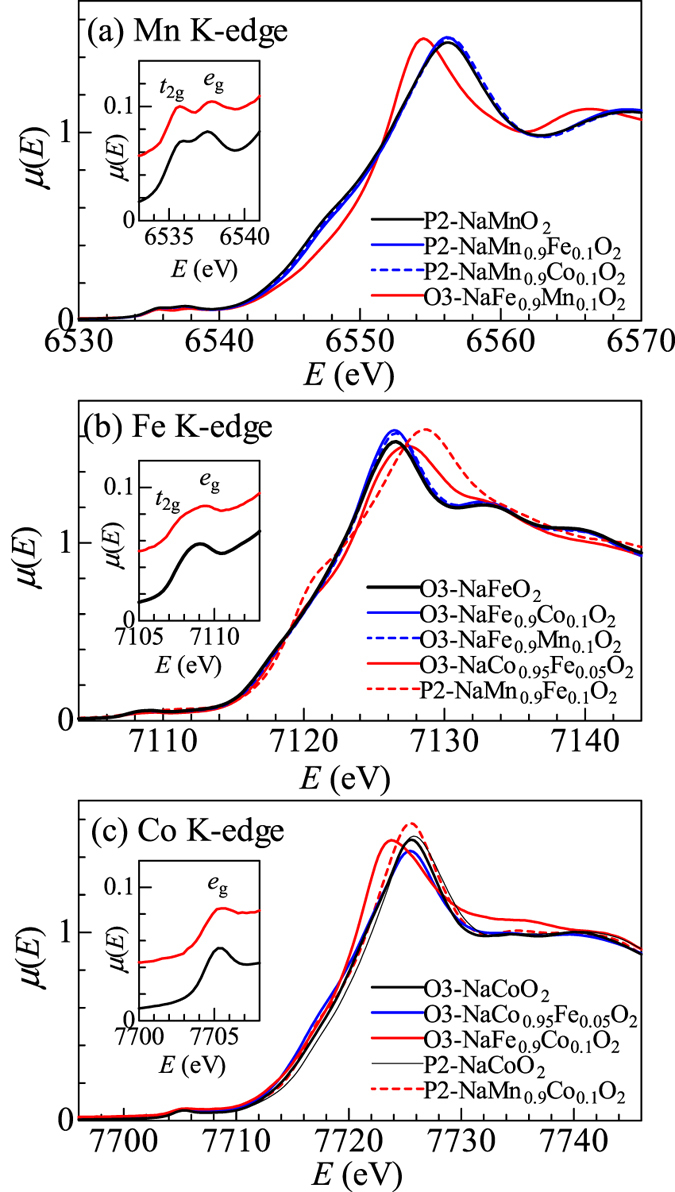
XANES spectra of pure and mixed layered oxides around the (**a**) Mn K-edge, (**b**) Fe K-edge, and (**c**) Co K-edge. The black curve represents the spectra of pure layered oxides. The blue and red curves correspond to the spectra of the majority and minority *M*s in the mixed layered oxides, respectively. The insets show magnified spectra in the pre-edge region.

Now, let us discuss the valence states of the minority *M*s and their relation to the interatomic distance, *i.e., d*_*M*-O_ and *d*_*M-M*_. Figure 4 shows the XANES spectra of the pure and mixed layered oxides around the (a) Mn K-edge, (b) Fe K-edge, and (c) Co K-edge. The black curve represents the spectra of the pure layered oxides. The blue and red curves correspond to the spectra of the majority and minority *M*s in the mixed layered oxides, respectively. The main peak is attributable to the 1s–4p transition. Their peak shifts are crude measures of the valence change: the valence increases or decreases if the peak shows a blue or red shift, respectively.

In the Mn K-edge spectra [Fig. 4(a)], the main peak of P2-NaMnO_2_ (black curve) is located at 6556.0 eV. We observed no detectable shift in the majority Mn spectrum (blue curves) in P2-NaMn_0.9_Fe_0.1_O_2_, indicating that the valence state of Mn is unchanged. In the minority Mn spectrum (red curve) in O3-NaFe_0.9_Mn_0.1_O_2_, the peak (=6554.0 eV) shows a significant red shift, indicating a decrease in the Mn valence from Mn^3+^ (*t*_2g_^3^*e*_g_^1^) with high-spin (HS) configuration. A trace of the valence change is also discernible in the pre-edge spectra [inset of Fig. 4(a)], which is attributable to the 1s–3d transition. The P2-NaMnO_2_ (black curve) spectrum shows a two-peak feature, whose higher- and lower-lying components are attributable to the 1s–3d*t*_2g_ and 1s–3 d*e*_g_ transitions, respectively. The relative intensity of the higher peak decreases in O3-NaFe_0.9_Mn_0.1_O_2_ (red curve), suggesting the partial formation of the *e*_g_-electron. Thus, the XANES spectrum indicates that the valence of the minority Mn approaches the divalent HS configuration (*t*_2g_^3^*e*_g_^2^). Such a valence change reduces the local lattice distortion around Mn, because the *r* value increases from *r* = 0.550 Å for Mn^3+^ to 0.780 Å for Mn^2+^. Here, we consider the Na deficiency effect on the Mn valance in O3-NaFe_0.9_Mn_0.1_O_2,_ whose actual chemical formula is Na_0.86_Fe_0.9_Mn_0.1_O_2_ (Table 2). The Na deficiency tends to increases the Mn valence to compensate the decrease in Na^+^. This trend is opposite to reduction of the Mn valence as suggested by the red shift of the main peak.

In the Fe K-edge spectra [Fig. 4(b)], the main peak of O3-NaFeO_2_ (black curve) is located at 7126.5 eV. We observed no detectable shift of the majority Fe spectrum (blue curves) in O3-NaFe_0.9_Co_0.1_O_2_. The peak (=7127.5 eV) in the minority Fe spectrum (red curve) in O3-NaCo_0.95_Fe_0.05_O_2_ shows a significant blue shift, suggesting that the Fe valence increases from Fe^3+^ (*t*_2g_^3^*e*_g_^2^). The pre-edge spectrum [black curve in the inset of Fig. 4(b)] of O3-NaFeO_2_ is rather broad, suggesting a two-peak feature consistent with HS Fe^3+^ state (*t*_2g_^3^*e*_g_^2^)^12^. The spectral weight at the high-energy side increases slightly in O3-NaCo_0.95_Fe_0.05_O_2_ (red curve), implying the partial formation of the *e*_g_-hole. Thus, the XANES spectrum implies that the valence of the minority Fe approaches the tetravalent HS configuration. Such a valence change reduces the local lattice distortion around Fe, because the *r* value decreases from *r* = 0.645 Å for Fe^3+^ to 0.585 Å for Fe^4+^. On the other hand, Ménétrier *et al*.^13^ performed ^57^Fe Mössbauer spectroscopy in Li-overstoichiometric Li_1.1_Co_1*y*_Fe_*y*_O_2_ (*y* < 0.08), in which Fe occupies the square-pyramidal site. This spectroscopy revealed that Fe at the square-pyramidal site takes the trivalent HS configuration (*t*_2g_^3^*e*_g_^2^). Another possible explanation for the unexpected XANES spectrum is that the spectral change is originated in the impurity phase. A close inspection of the XRD pattern [Fig. S2(b)] reveals weak additional reflections that are unaccounted by the trigonal model. We performed two-phase Rietveld analysis with adding spinel phase (*Fd-3m*; *Z* = 8). The additional reflections are well reproduced by 5 wt% spinel phase with *a* = 8.0805(2) Å (Fig. S14). Judging from the cell parameter, the spinel phase is considered to be Fe-substituted Co_3_O_4_. Then, the tetrahedrally-coordinated Fe in the impurity phase may be responsible for the XANES spectrum. We further observed a significant blue shift in the P2-NaMn_0.9_Fe_0.1_O_2_ spectrum (broken red curve), suggesting increases in the Fe valence. We note that P2-NaMnO_2_ does not show any cooperative Jahn-Teller distortion of HS Mn^3+^. The valence change, however, may be due to the Na deficiency effect in P2-NaMn_0.9_Fe_0.1_O_2_ (*g* = 0.31).

Thus, the minority elements adjust their valences so that their ionic radii approach that of the majority *M*. This empirical relation is effective in the design of functional materials with such a partial substitution because we can predict the valence change of the minority *M*s. Specifically, the valence state of the substituted *M* has a determining effect on the physical properties of the oxide, such as the redox voltage in the battery material, exchange interaction in magnetic material, and electron-lattice interaction in the dielectric material.

In the Co K-edge spectra [Fig. 4(c)], the main peak of O3-NaCoO_2_ (black curve) is located at 7725.5 eV. We note that the peak position of P2-NaCoO_2_ is almost the same as that of O3-NaCoO_2_. We observed no detectable peak shift of the majority Co spectrum (blue curve) in O3-NaCo_0.95_Fe_0.05_O_2_. The peak (=7724.0 eV) in the minority Co (red curve) in O3-NaFe_0.9_Co_0.1_O_2_ shows a significant red shift, indicating that the Co valence decreases from Co^3+^ (*t*_2g_^6^). The pre-edge spectra of O3-NaFe_0.9_Co_0.1_O_2_ [red curve in the inset of Fig. 4(c)], however, remain with a single peak with no lower-lying absorption corresponding to the 1s–3d*t*_2g_ transition. In addition, the *d*_Co-*M*_ value of O3-NaFe_0.9_Co_0.1_O_2_ is much smaller than the mean-field value [Fig. 3(b)], which is curious because Co^3+^ (*t*_2g_^6^) has the smallest ionic radius (=0.550 Å). These observations imply that Co^3+^ takes the ligand-hole state (*t*_2g_^6^*e*_g_^1^*L*, where *L* means the ligand hole) rather than the trivalent LS state (*t*_2g_^6^*e*_g_^0^). Due to the complicated ligand-hole state of the Co ion, it seems to be difficult to put forth a simplified argument based only on the ionic radius.

## Summary

The systematic EXAFS analyses revealed that the local interatomic distance (*d*_*M*-O_) around the minority *M* approaches that around the majority *M* to reduce the local lattice distortion. The valence of the minority Mn changes so that its ionic radius approaches that of the majority *M*. This empirical relation is effective in the design of functional materials with such a partial substitution.

## Method

### Sample preparation

Three pure and five mixed layered oxides were prepared from Na_2_O_2_, Na_2_CO_3_, Co_3_O_4_, Fe_2_O_3_, and Mn_3_O_4_ by solid-state reaction. In Fe-based O3-type compounds, Na_2_O_2_, Fe_2_O_3_, and the minority *M* sources were mixed in a 1.2: 1 − *x: x* atomic ratio and calcined at 943 K for 24 h in air. In the Co-based O3-type compounds, Na_2_O_2_, Co_3_O_4_, and the minority *M* sources were mixed in a 1.2: 1 − *x: x* atomic ratio and calcined at 823 K for 16 h in O_2_. In the Mn-based P2-type compounds, Na_2_CO_3_, Mn_3_O_4_, and the minority *M* sources were mixed in a 0.7: 1 − *x: x* atomic ratio and calcined at 1273 K for 24 h in air. The laboratory X-ray diffraction patterns show O3- or P2-type structure without detectable impurities.

### Crystal structure

To determine the crystal structure and Na percentage (*x*), we performed synchrotron-radiation X-ray powder diffraction measurements at the BL8A beamline of the Photon Factory, KEK. The samples were finely ground and placed in 0.3 mmϕ glass capillaries. The capillaries were sealed and mounted on the Debye-Scherrer camera. The powder diffraction patterns were detected with an imaging plate. The exposure time was 5–10 minutes. The wavelength (=0.689033 Å) of the X-ray was calibrated by the lattice constant of standard CeO_2_ powders. The diffraction patterns of the O3-type compounds were analyzed by the Rietveld method (Rietan-FP^14^: Figs S1 and S2) with a trigonal model (*R-*
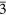
*m*; *Z* = 3, hexagonal setting). The diffraction patterns of the P2-type compounds were analyzed by the Rietveld method (Fig. S3) with a hexagonal model (*P*6_3_/mmc; *Z* = 2). We observed neither traces of impurities nor secondary phases. The obtained parameters, *i.e.*, lattice constant (*a* and *c*), *z* coordinate (*z*) of O, and Na percentage (*x*), are listed in Table 2.

### X-ray absorption spectroscopy

The X-ray absorption spectroscopy (XAS) measurements were conducted at BL-9C of the Photon Factory, KEK. The powder was finely ground, and mixed with BN, and pressed into pellets with 5 mm in diameter. The weight of the powder in the pellets was optimized by making the edge jump Δμt ≈ 1 for majority elements and 0.32–0.77 for minority elements. The storage ring was operated at 2.5 GeV and 450 mA, and the maximum photon flux was 1 × 10^11^ photons s^−1^. The XAS were recorded in a transmission mode with a Si(111) double-crystal monochromator at 300 K. N_2_ (100%) gas-filled ion camber was used to monitor the incident X-ray intensity and Ar (15%) and N_2_ (85%) gas-filled ion camber was used to monitor the transmitted intensity. The higher harmonics were eliminated by a detune to 60% of incident X-ray. The ratio of higher harmonics contamination estimated by the detection efficiency of the ion chambers was less than 2 × 10^−4^. The beam size at the sample position was 0.8 × 0.6  mm (h × w). The wavelengths of the monochromator were calibrated with the absorption edge of Fe, Mn, and Co foil. In X-ray near edge structure (XANES) analyses, the background subtraction and normalization were performed using the ATHENA program^15^.

### EXAFS analysis

The extended X-ray fine structure (EXAFS) analyses were performed using the ATHENA and ARTEMIS programs^15^. First, the oscillatory components were extracted using the ATHENA program after background subtraction and normalization of the absorption spectra. Thus, we obtained *χ*(*k*)*k*^3^−*k* plots, where *χ* and *k* are the oscillatory components of the normalized absorption and angular wavenumber, respectively. Then, we performed Fourier transformation of *χ*(*k*)*k*^3^ in the *k*-range from 2 Å^−1^ to 11.2 Å^−1^.

The plots thus obtained FT[*χ*(*k*)*k*^3^]−*R* were analyzed using the ATHENA program (Figs S4–S11). In the plane wave and single-scattering approximation, *χ*(*k*) around the K-edge is expressed by the EXAFS equation as:


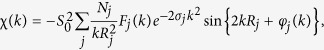


where *S*_0_, *N*_j_, *R*_j_, *F*_j_, *σ*_j_^2^, and *φ*_j_ are the passive electron reduction factor, degeneracy of path, path length, effective scattering amplitude, mean square displacement, and effective scattering phase shift of the *j*th atom, respectively. The angular wavelength *k* is defined by 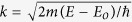 where *m, E*, and *E*_0_ are the electron mass, energy of the incident X-ray, and energy shift, respectively. In the usual EXAFS analysis, *S*_0_, *N*_j_, *σ*_j_^2^, *E*_0_, and *R*_j_ are adjusted to minimize the difference between the experimental data and EXAFS equation. In the O3 compound, we included the contribution from the first- (O), second- (*M*), and third- (Na) nearest neighbor elements. In the P2 compounds, we included the contributions from the first- (O), third- (*M*), and fifth- (O) nearest neighbor elements. We omitted the contribution from the second- (Na2) and fourth- (Na1) nearest neighbor elements because the occupancy (*g* = 0.21–0.41) is small. In both cases, we fixed *N*_j_ for the three elements at the crystallographic value (=6). We used the same *S*_0_ and *E*_0_ values for the three elements. The least-squares fittings were performed in the *R* range from 1 Å to 3 Å.

The details of the analytical results are shown in Figs S4–S11. We found that the R values of the third paths, *i.e*. the *M*–Na distances in the O3 compounds and the longer *M* - O distances of the P2 compounds, are seriously deviated from the crystallographic values. In addition, the* σ*_j_^2^ values for these paths are significantly large (~0.01–0.03 Å^2^) as compared with those of the first and second paths. In this sense, the EXAFS analysis model including the third paths remains ambiguous. We performed another EXAFS analysis with omitting the third path to validate the discussion above. We observed the same trend of the *M* – O and *M* – *M* distances between the compounds even if we omitted the third path. (Figs S12 and S13) Thus, we confirmed that the *R* value of the *M* – O and *M* – *M* distances are reliable even though those of the third path remains ambiguous.

## Additional Information

**How to cite this article:** Akama, S. *et al*. Local structures around the substituted elements in mixed layered oxides. *Sci. Rep.* **7**, 43791; doi: 10.1038/srep43791 (2017).

**Publisher's note:** Springer Nature remains neutral with regard to jurisdictional claims in published maps and institutional affiliations.

## Supplementary Material

Supplemental Information

## Figures and Tables

**Table 1:** Interatomic distances in layered oxides.

Compound	Majority *d*_*M*-O_ (Å)	Minority *d*_*M*-O_ (Å)	Majority *d*_*M*-*M*_ (Å)	Minority *d*_*M*-*M*_ (Å)
O3-NaFeO_2_	2.041 (11)	—	3.039 (8)	—
O3-NaFe_0.9_Mn_0.1_O_2_	2.033 (8)	1.930 (9)	3.022 (6)	2.997 (8)
O3-NaFe_0.9_Co_0.1_O_2_	2.037 (8)	1.886 (9)	3.031 (6)	2.858 (12)
O3-NaCoO_2_	1.905 (6)	—	2.851 (6)	—
O3-NaCo_0.95_Fe_0.05_O_2_	1.910 (6)	2.009 (10)	2.858 (12)	2.915 (9)
P2-NaMnO_2_	1.907 (8)	—	2.898 (9)	—
P2-NaMn_0.9_Fe_0.1_O_2_	1.908 (6)	1.998 (12)	2.901 (6)	2.905 (11)
P2-NaMn_0.9_C_0.1_O_2_	1.909 (7)	1.914 (9)	2.880 (7)	2.848 (8)

The values were obtained using EXAFS analyses.

**Table 2:** Lattice constants (*a* and *c*), *z* coordinate (*z*) of O, and Na percentage (*x*) of layered oxides.

Compound	*a* (Å)	*c* (Å)	*z*	*x*
O3-NaFeO_2_	3.02157 (5)	16.07402 (31)	0.23298 (12)	0.9831 (45)
O3-NaFe_0.9_Mn_0.1_O_2_	3.01862 (12)	16.15361 (65)	0.23249 (21)	0.8632 (77)
O3-NaFe_0.9_Co_0.1_O_2_	3.01954 (7)	16.08690 (40)	0.23275 (14)	0.9771 (53)
O3-NaCoO_2_	2.89022 (4)	15.60949 (24)	0.22948 (11)	0.9624 (43)
O3-NaCo_0.95_Fe_0.05_O_2_	2.89368 (9)	15.63520 (66)	0.22982 (21)	0.9572 (71)
P2-NaMnO_2_	2.87337 (8)	11.16667 (57)	0.08771 (37)	0.6734 (169)
P2-NaMn_0.9_Fe_0.1_O_2_	2.87730 (7)	11.19772 (44)	0.08559 (27)	0.7218 (141)
P2-NaMn_0.9_C_0.1_O_2_	2.86177 (13)	11.19690 (84)	0.07971 (34)	0.4443 (163)

In the O3-type compounds, the atomic coordinates were Na (0,0,0), *M* (0,0,1/2), and O (0,0, *z*).

The *x* value is the occupancy at the Na sites. In the P2-type compounds, the atomic coordinates were Na1 (1/3,2/3,3/4), Na2 (0,0,1/4), *M* (0,0,0), and O (1/3,2/3, *z*). The *x* value is the sum of the occupancies at the Na1 and Na2 sites.
