# Supplementary material for: Local structures around the substituted elements in mixed layered oxides
Source: Sci Rep. 2017 Mar 2;7:43791. doi: 10.1038/srep43791 (PMC5333154; doi:10.1038/srep43791)
Supplement: Supplemental Information [file srep43791-s1.pdf]

Supporting information

Local structures around the substituted elements in mixed layered oxides

Shota Akama[1], Wataru Kobayashi[1-4], Kaoru Amaha[1], Hideharu Niwa[1-3], Hiroaki Nitani[5], and Yutaka Moritomo[1-4*]

[1]Graduate School of Pure and Applied Science, University of Tsukuba, Tsukuba 305-8571, Japan

[2]Faculty of Pure and Applied Science, University of Tsukuba, Tsukuba 305-8571, Japan

[3]Center for Integrated Research in Fundamental Science and Engineering (CiRfSE), University Tsukuba, Tsukuba 305-8571, Japan

[4]Tsukuba Research Center for Interdisciplinary Materials Sciences (TIMS), University of Tsukuba, Tsukuba 305-8571, Japan

[5]Institute of Materials Science, High Energy Accelerator Research Organization (KEK), Tsukuba 305-0801, Japan

Contact information:

Yutaka Moritomo (Professor)

Faculty of Pure and Applied Science,

Univ. of Tsukuba, Tennodai 1-1-1, Tsukuba 305-8571, Japan

Tel & Fax +81-29-853-4337

e-mail: moritomo.yutaka.gf@u.tsukuba.ac.jp

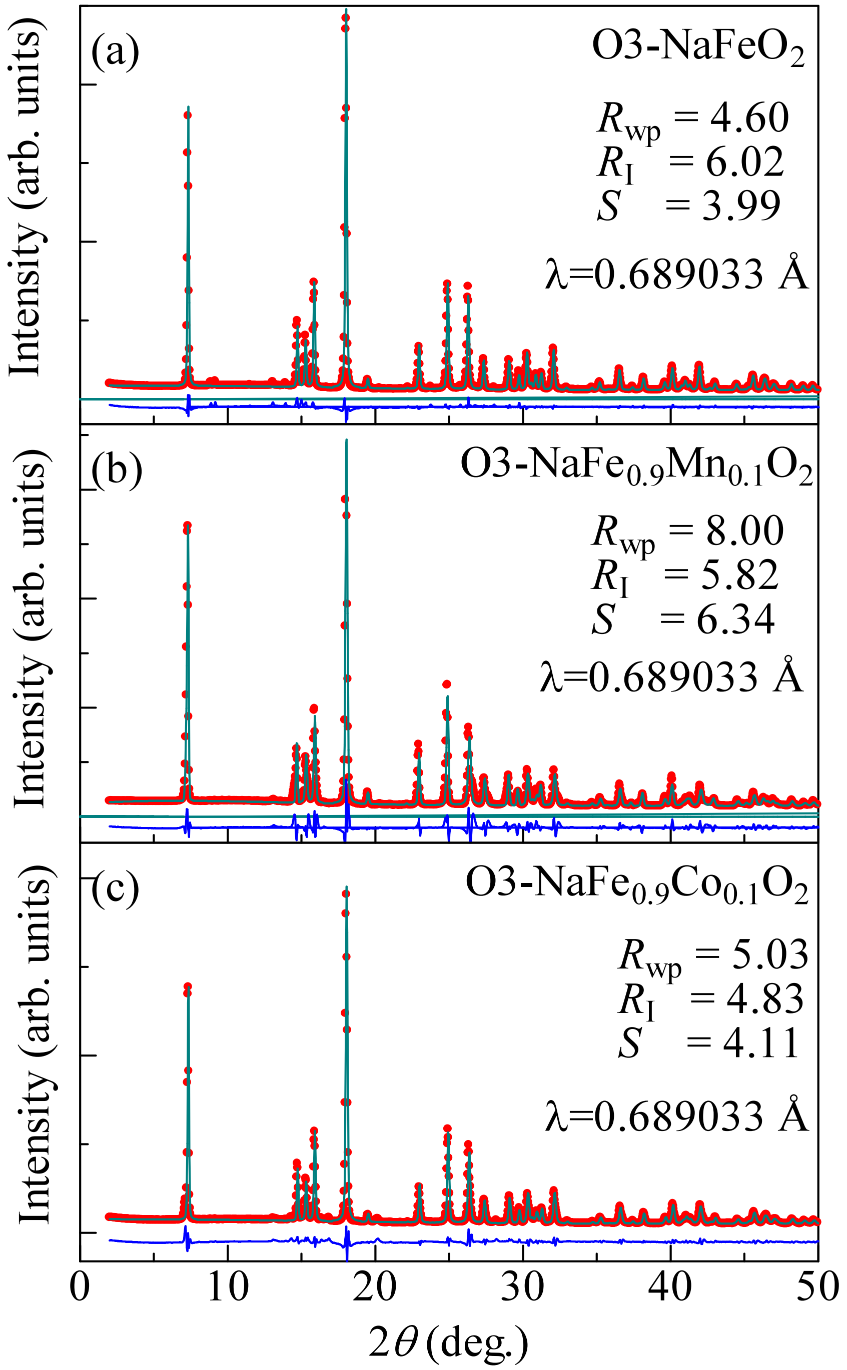

Fig. S1: Synchrotron-radiation X-ray powder diffraction pattern of (a) O3-$NaFeO_2$, (b) O3-$NaFe_{0.9}Mn_{0.1}O_2$, and (c) O3-$NaFe_{0.9}Co_{0.1}O_2$. The green curve is result of Rietveld refinement with a trigonal model ($R\bar{3}m$; Z= 3, hexagonal setting). The wavelength (=0.689033 Å) of the X-ray was calibrated by the lattice constant of standard CeO_2 powders. The blue curve is the difference between experimental data and calculation. Obtained structural parameters are listed in Tables S1, S2, and S3.

atom	site	g	x	y	z	B
Na	3a	0.9831(45)	0	0	0	0.661(49)
M	3b	1	0	0	1/2	0.324(15)
O	6c	1	0	0	0.23298(12)	0.542(41)

Table S1: Atomic coordinates (*x*, *y*, *z*), occupancy (*g*) and atomic displacement parameters (*B*) of the green phase of O3-$NaFeO_2$ at 300 K. The crystal structure is hexagonal model (*R*3*m*; *Z*= 3) with *a* = 3.02157(5) Å and c = 16.07402(31) Å. R_{wp}, R_I, and *S* are 4.60%, 6.02%, and 3.99, respectively. The *M* - O, *M* - *M*, and *M* - Na distances are 2.04403 Å, 3.02157 Å, and 3.19631 Å, respectively.

atom	site	g	x	y	z	B
Na	3a	0.8632(77)	0	0	0	0.568(100)
M	3b	1	0	0	1/2	0.499(35)
O	6c	1	0	0	0.23249(21)	0.687(78)

Table S2: Atomic coordinates (*x*, *y*, *z*), occupancy (*g*) and atomic displacement parameters (*B*) of the green phase of O3-$NaFe_{0.9}Mn_{0.1}O_2$ at 300 K. The crystal structure is hexagonal model (*R*3*m*; *Z*= 3) with *a* = 3.01862(12) Å and c = 16.15361(65) Å. R_{wp}, R_I, and *S* are 8.00%, 5.82%, and 6.34, respectively. The *M* - O, *M* - *M*, and *M* - Na distances are 2.04147 Å, 3.01862 Å, and 3.20713 Å, respectively.

atom	site	g	x	y	z	B
Na	3a	0.9771(53)	0	0	0	0.519(62)
M	3b	1	0	0	1/2	0.245(26)
O	6c	1	0	0	0.23275(14)	0.428(50)

Table S3: Atomic coordinates (*x*, *y*, *z*), occupancy (*g*) and atomic displacement parameters (*B*) of the green phase of O3-$NaFe_{0.9}Co_{0.1}O_2$ at 300 K. The crystal structure is hexagonal model (*R*3*m*; *Z*= 3) with *a* = 3.01954(7) Å and c = 16.08690(40) Å. R_{wp}, R_I, and *S* are 5.03%, 4.83%, and 4.11, respectively. The *M* - O, *M* - *M*, and *M* - Na distances are 2.04187 Å, 3.01954 Å, and 3.19809 Å, respectively.

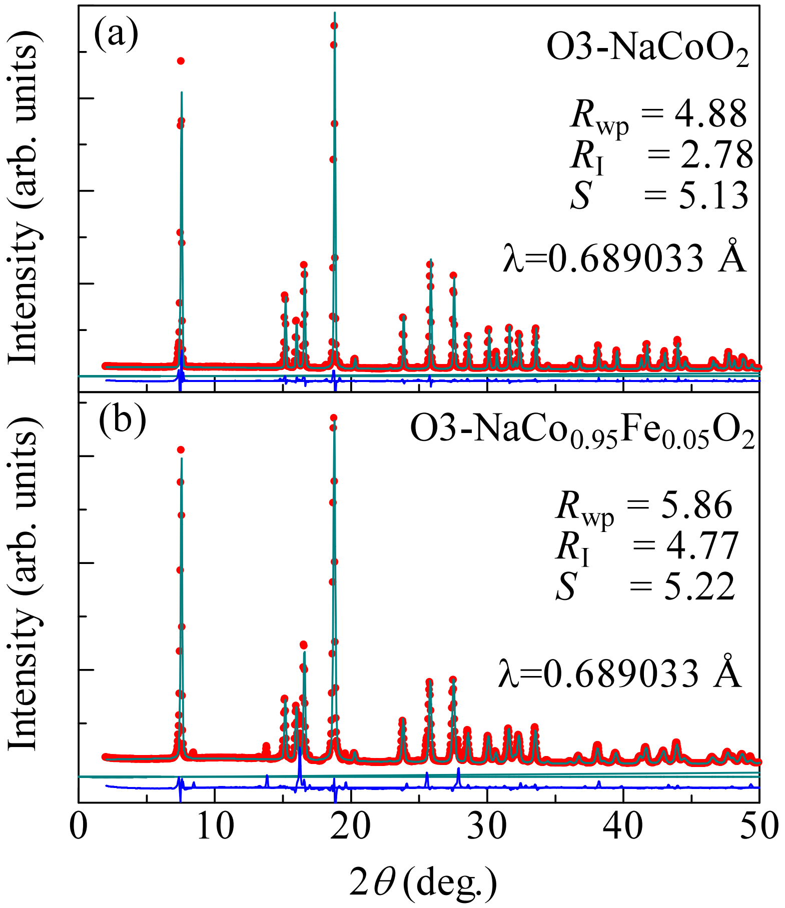

Fig. S2: Synchrotron-radiation X-ray powder diffraction pattern of (a) O3-$NaCoO_2$, (b) and O3-$NaCo_{0.95}Fe_{0.05}O_2$. The green curve is result of Rietveld refinement with a trigonal model ($R\bar{3}m$; Z= 3, hexagonal setting). The blue curve is the difference between experimental data and calculation. The wavelength (=0.689033 Å) of the X-ray was calibrated by the lattice constant of standard CeO_2 powders. Obtained structural parameters are listed in Tables S4 and S5.

atom	site	g	x	y	z	B
Na	3a	0.9624(43)	0	0	0	0.271(36)
M	3b	1	0	0	1/2	0.011(13)
O	6c	1	0	0	0.22948(11)	0.227(32)

Table S4: Atomic coordinates (x, y, z), occupancy (g) and atomic displacement parameters (B) of the green phase of O3- $NaCoO_2$ at 300 K. The crystal structure is hexagonal model ($R3m$; Z= 3) with a = 2.89022(4) Å and c =15.60949(24) Å. R_{wp}, R_I, and S are 4.88%, 2.78%, and 5.13, respectively. The M - O, M - M, and M - Na distances are 1.93547 Å, 2.89022 Å, and 3.09074 Å, respectively.

atom	site	g	x	y	z	B
Na	3a	0.9572(71)	0	0	0	0.333(81)
M	3b	1	0	0	1/2	0.074(33)
O	6c	1	0	0	0.22982(21)	0.279(62)

Table S5: Atomic coordinates (x, y, z), occupancy (g) and atomic displacement parameters (B) of the green phase of O3-$NaCo_{0.95}Fe_{0.05}O_2$ at 300 K. The crystal structure is hexagonal model ($R3m$; Z= 3) with a = 2.89368(9) Å and c =15.63520(66) Å. R_{wp}, R_I, and S are 5.86%, 4.77%, and 5.22, respectively. The M - O, M - M, and M - Na distances are 1.94063 Å, 2.89368 Å, and 3.09543 Å, respectively.

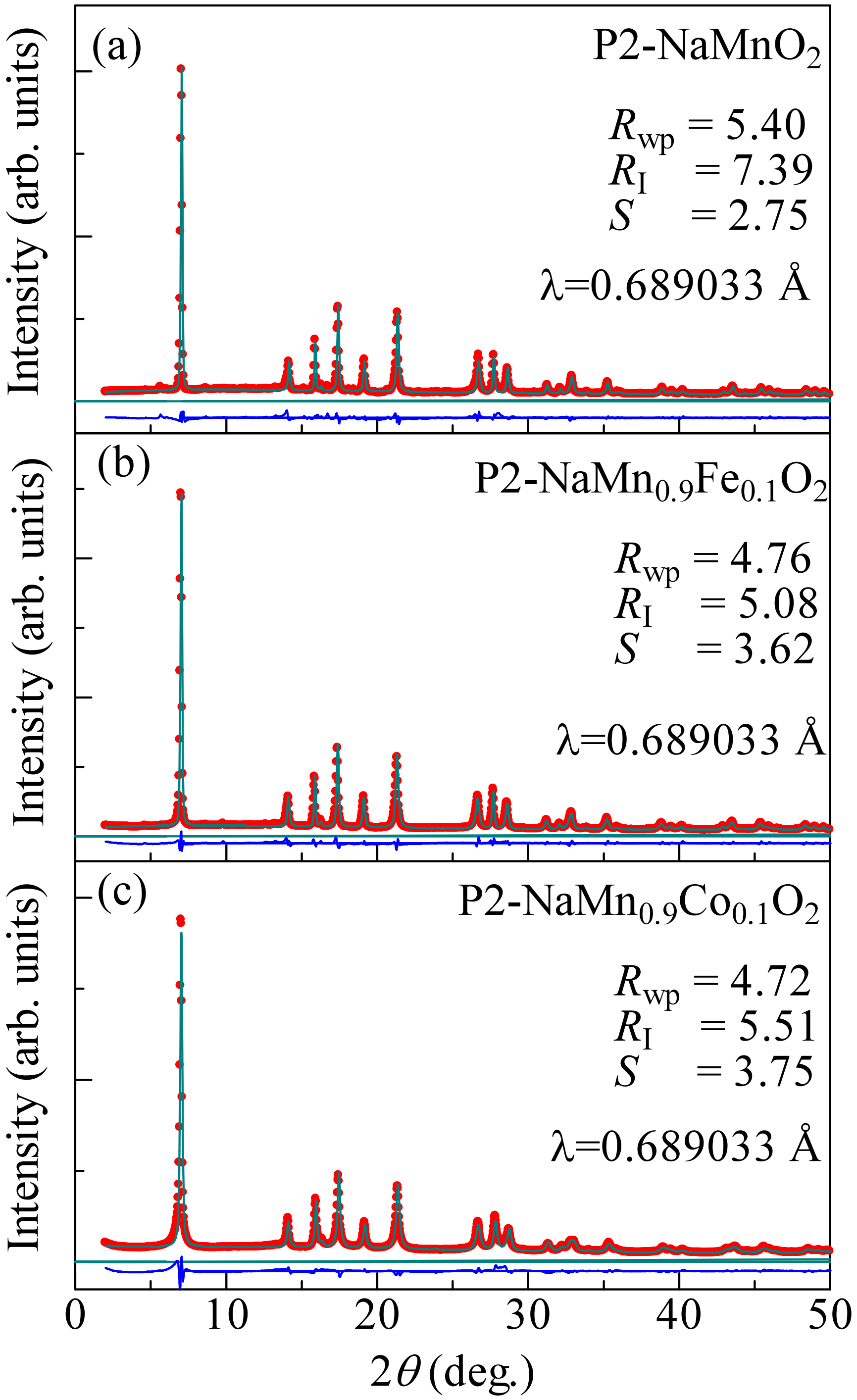

Fig. S3: Synchrotron-radiation X-ray powder diffraction pattern of (a) P2-$NaMnO_2$, (b) P2-$NaMne_{0.9}Fe_{0.1}O_2$, and (c) P2-$NaMn_{0.9}Co_{0.1}O_2$. The green curve is result of Rietveld refinement with a hexagonal model ($P6_3$/mmc; Z= 2). The wavelength (=0.689033 Å) of the X-ray was calibrated by the lattice constant of standard CeO_2 powders. The blue curve is the difference between experimental data and calculation. Obtained structural parameters are listed in Tables S6, S7, and S8.

atom	site	g	x	y	z	B
Na1	2d	0.3979(95)	1/3	2/3	3/4	6.739(460)
Na2	2b	0.2745(74)	0	0	1/4	6.739
M	2a	1	0	0	0	0.621(38)
O	4f	1	1/3	2/3	0.08771(37)	0.414(73)

Table S6: Atomic coordinates (*x*, *y*, *z*), occupancy (*g*) and atomic displacement parameters (*B*) of the green phase of P2-$NaMnO_2$ at 300 K. The crystal structure is hexagonal model (*R*3*m*; *Z*= 3) with *a* = 2.87337(8) Å and c =11.16667(57) Å. R_{wp}, R_I, and *S* are 5.40%, 7.39%, and 2.75, respectively. The *M* - O, *M* - *M*, and *M* - Na distances are 1.92652 Å, 2.87337 Å, and 2.79168 Å, respectively.

atom	Site	g	x	y	z	B
Na1	2d	0.4140(78)	1/3	2/3	3/4	7.263(361)
Na2	2b	0.3078(63)	0	0	1/4	7.263
M	2a	1	0	0	0	0.460(29)
O	4f	1	1/3	2/3	0.08559(27)	0.198(27)

Table S7: Atomic coordinates (*x*, *y*, *z*), occupancy (*g*) and atomic displacement parameters (*B*) of the green phase of P2- $NaMn_{0.9}Fe_{0.1}O_2$ at 300 K. The crystal structure is hexagonal model (*R*3*m*; *Z*= 3) with *a* = 2.87730(7) Å and c =11.19772(44) Å. R_{wp}, R_I, and *S* are 4.76%, 5.08%, and 3.62, respectively. The *M* - O, *M* - *M*, and *M* - Na distances are 1.91748 Å, 2.87730 Å, and 2.79942 Å, respectively.

atom	site	g	x	y	z	B
Na1	2d	0.2360(74)	1/3	2/3	3/4	2.875(429)
Na2	2b	0.2083(89)	0	0	1/4	2.875
M	2a	1	0	0	0	0.532(36)
O	4f	1	1/3	2/3	0.07971(34)	0.532

Table S8: Atomic coordinates (*x*, *y*, *z*), occupancy (*g*) and atomic displacement parameters (*B*) of the green phase of P2- $NaMn_{0.9}Co_{0.1}O_2$ at 300 K. The crystal structure is hexagonal model (*R*3*m*; *Z*= 3) with *a* = 2.86177(13) Å and c =11.19690(84) Å. R_{wp}, R_I, and *S* are 4.72%, 5.51%, and 3.75, respectively. The *M* - O, *M* - *M*, and *M* - Na distances are 1.87793 Å, 2.86177 Å, and 2.79923 Å, respectively.

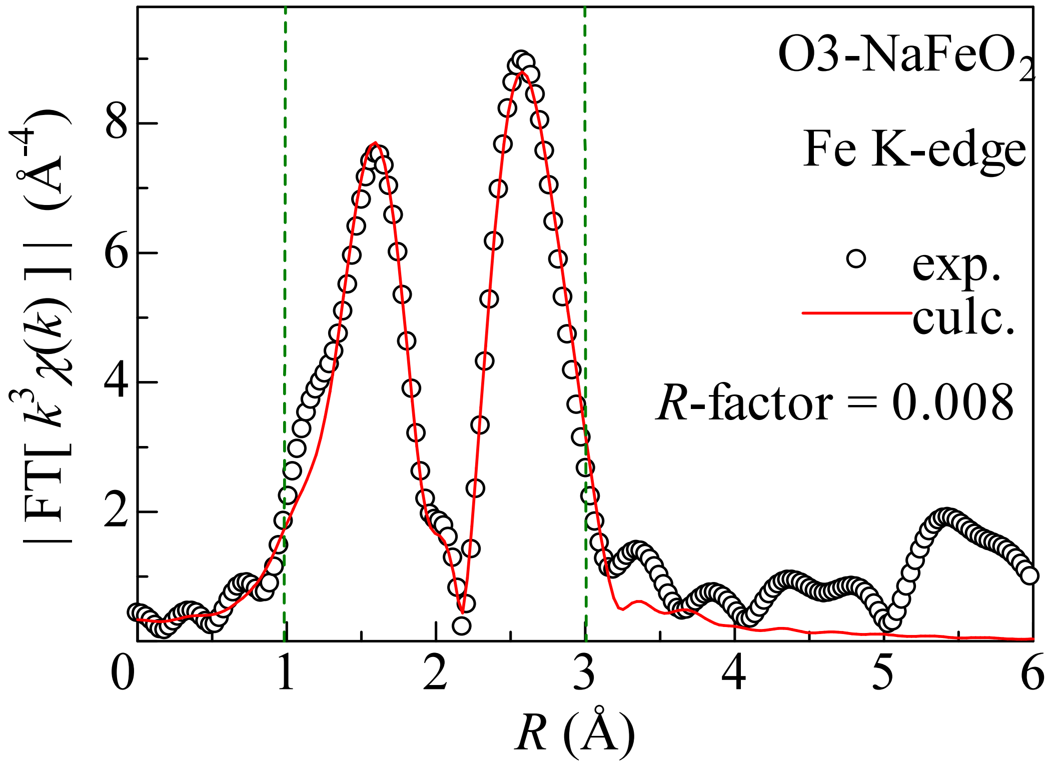

Fig. S4: FT[$\chi(k)k^3$]–R plot of Fe K-edge of O3-$NaFeO_2$. Red curve is the least-squares fitting with the EXFAS equation in the R range from 1Å to 3Å. The obtained parameters are the following. S_0, N_j, R_j, σ_j^2, E_0, are the passive electron reduction factor, degeneracy of path, path length, mean square displacement, energy shift, respectively.

Path	S_0^2	N_j	R (Å)	$\sigma^2(10^{-3}$ Å$^2)$	E_0 (eV)
Fe-O	0.69(10)	6	2.041(11)	6.0(15)	7121.8(17)
Fe-Fe	0.69	6	3.039(8)	5.6(10)	7121.8
Fe-Na	0.69	6	3.076(48)	17.8(123)	7121.8

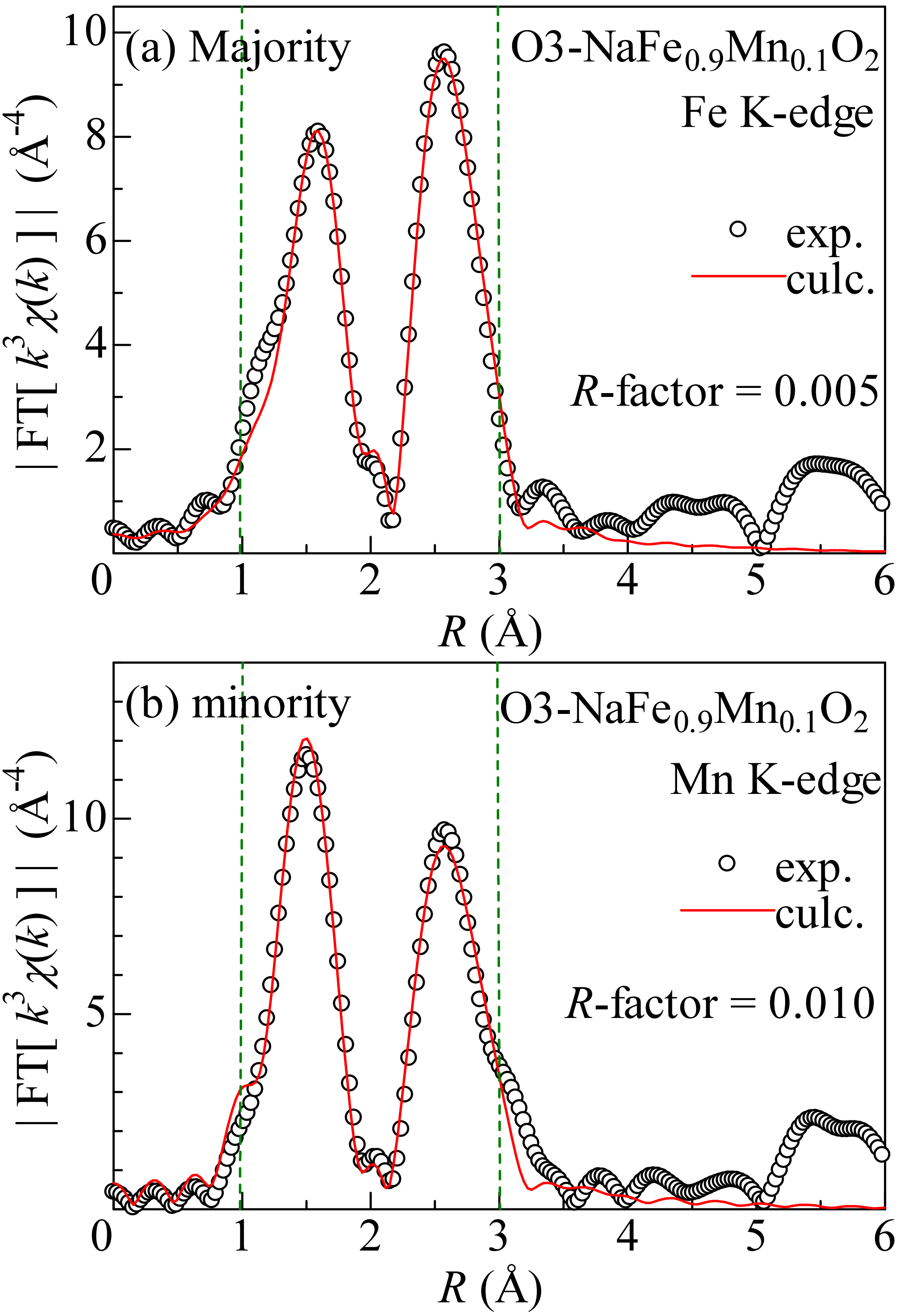

Fig. S5: FT[$\chi(k)k^3$]–R plots of (a) Fe K-edge and (b) Mn K-edge of O3- $NaFe_{0.9}Mn_{0.1}O_2$.. Red curve is the least-squares fitting with the EXFAS equation in the R range from 1Å to 3Å. The obtained parameters are the followings.

majority Fe

Path	S_0^2	N_j	R (Å)	$\sigma^2 (10^{-3}$ Å$^2)$	E_0 (eV)
Fe-O	0.69	6	2.033(8)	5.6(6)	7121.6(12)
Fe-TM	0.69	6	3.022(6)	5.7(4)	7121.6
Fe-Na	0.69	6	3.022(38)	23.4(121)	7121.6

minority Mn

Path	S_0^2	N_j	R (Å)	$\sigma^2 (10^{-3}$ Å$^2)$	E_0 (eV)
Mn-O	0.73	6	1.930(9)	4.6(7)	6547.9(14)
Mn-TM	0.73	6	2.997(8)	6.1(6)	6547.9
Mn-Na	0.73	6	3.041(51)	22.0(133)	6547.9

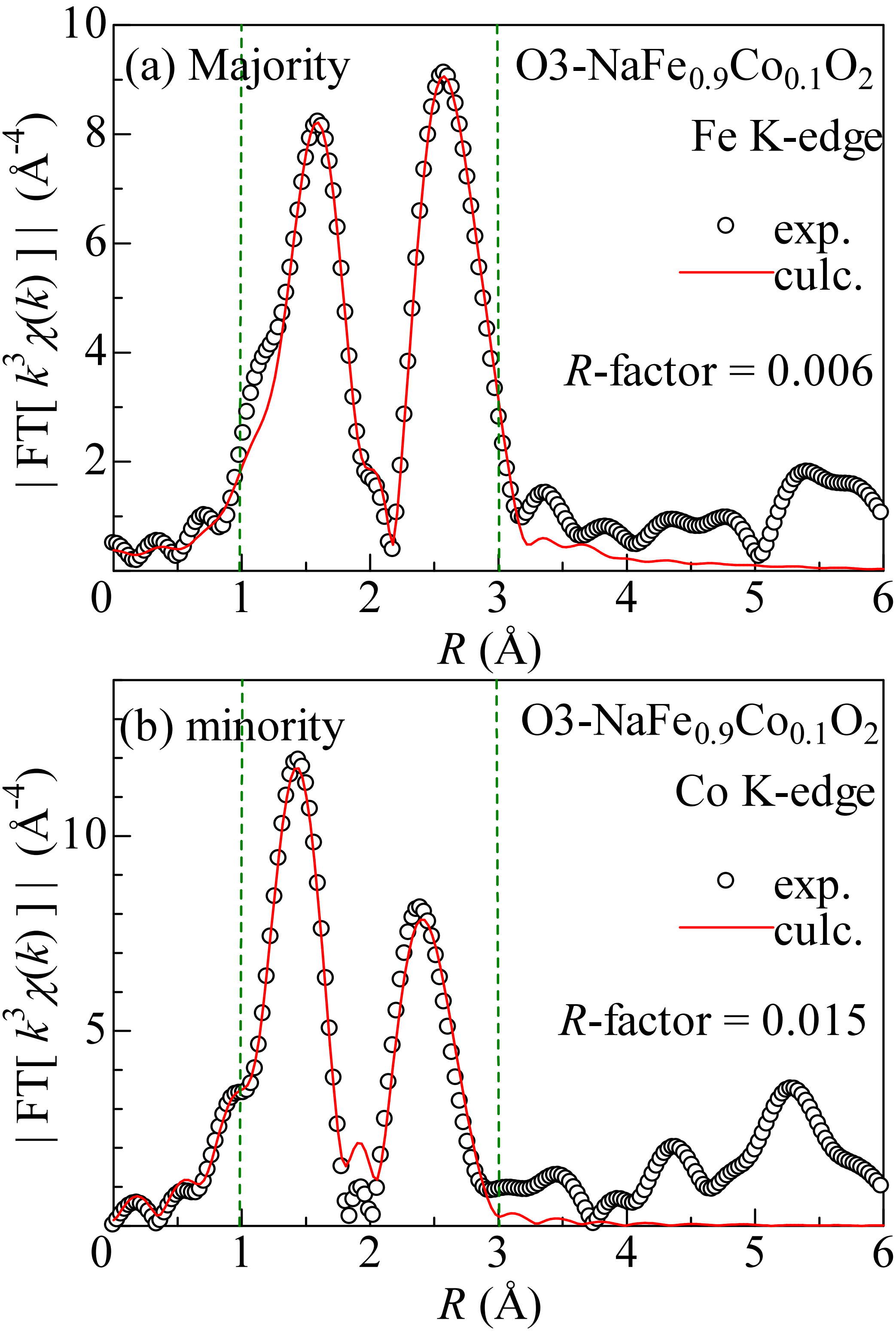

Fig. S6: FT[$\chi(k)k^3$]–R plots of (a) Fe K-edge and (b) Co K-edge of O3-$NaFe_{0.9}Co_{0.1}O_2$. Red curve is the least-squares fitting with the EXFAS equation in the R range from 1Å to 3Å. The obtained parameters are the followings.

majority Fe

Path	S_0^2	N_j	R (Å)	$\sigma^2 (10^{-3}$ Å$^2)$	E_0 (eV)
Fe-O	0.69	6	2.037(8)	5.4(6)	7121.6(13)
Fe-TM	0.69	6	3.031(6)	5.7(4)	7121.6
Fe-Na	0.69	6	3.059(38)	22.3(121)	7121.6

minority Co

Path	S_0^2	N_j	R (Å)	$\sigma^2 (10^{-3}$ Å$^2)$	E_0 (eV)
Co-O	0.77	6	1.886(9)	4.7(7)	7711.9(17)
Co-TM	0.77	6	2.858(12)	9.9(10)	7711.9
Co-Na	0.77	6	3.269(138)	29.1(211)	7711.9

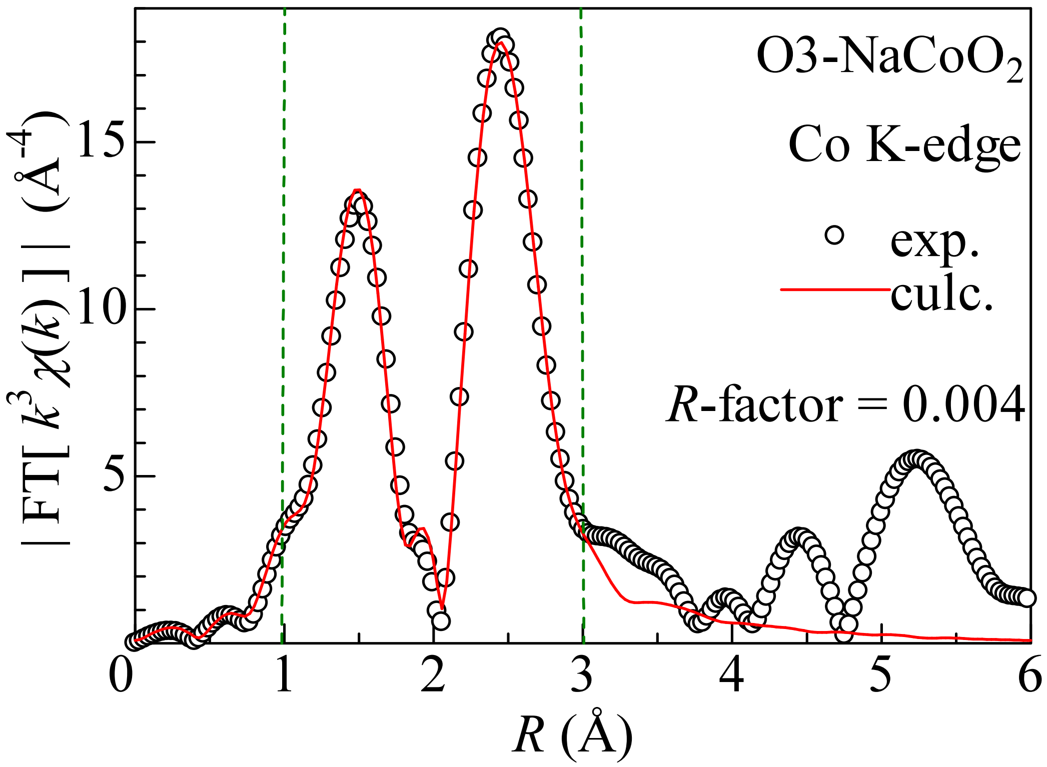

Fig. S7: FT[$\chi(k)k^3$]–R plot of Co K-edge of O3-$NaCoO_2$. Red curve is the least-squares fitting with the EXFAS equation in the R range from 1Å to 3Å. The obtained parameters are the following.

path	S_0^2	N_j	R (Å)	σ^2(10^{-3} Å^2)	E_0 (eV)
Co-O	0.77(7)	6	1.905(6)	3.6(9)	7716.8(11)
Co-Co	0.77	6	2.851(6)	3.0(6)	7716.8
Co-Na	0.77	6	3.382(59)	16.4(97)	7716.8

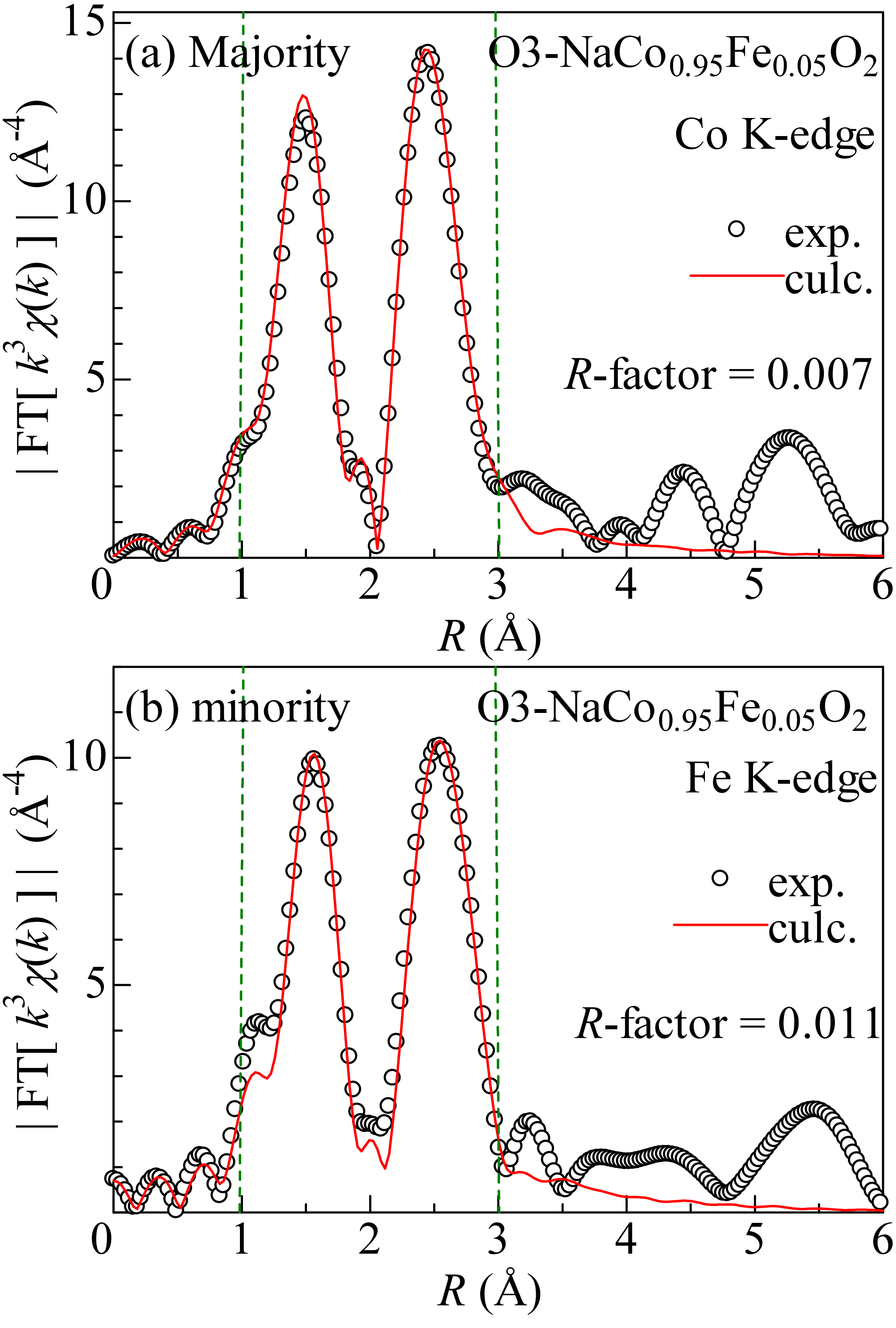

Fig. S8: FT[$\chi(k)k^3$]–R plots of (a) Co K-edge and (b) Fe K-edge of O3-$NaCo_{0.95}Fe_{0.05}O_2$. Red curve is the least-squares fitting with the EXFAS equation in the *R* range from 1Å to 3Å. The obtained parameters are the followings.

majority Co

Path	S_0^2	N_j	R (Å)	$\sigma^2 (10^{-3}$ Å$^2)$	E_0 (eV)
Co-O	0.77	6	1.910(6)	3.9(6)	7716.4(11)
Co-TM	0.77	6	2.853(6)	4.7(4)	7716.4
Co-Na	0.77	6	3.400(59)	18.2(106)	7716.4

minority Fe

Path	S_0^2	N_j	R (Å)	$\sigma^2 (10^{-3}$ Å$^2)$	E_0 (eV)
Fe-O	0.69	6	2.009(10)	4.1(7)	7121.7(17)
Fe-TM	0.69	6	2.915(9)	5.6(5)	7121.7
Fe-Na	0.69	6	3.269(142)	34.1(333)	7121.7

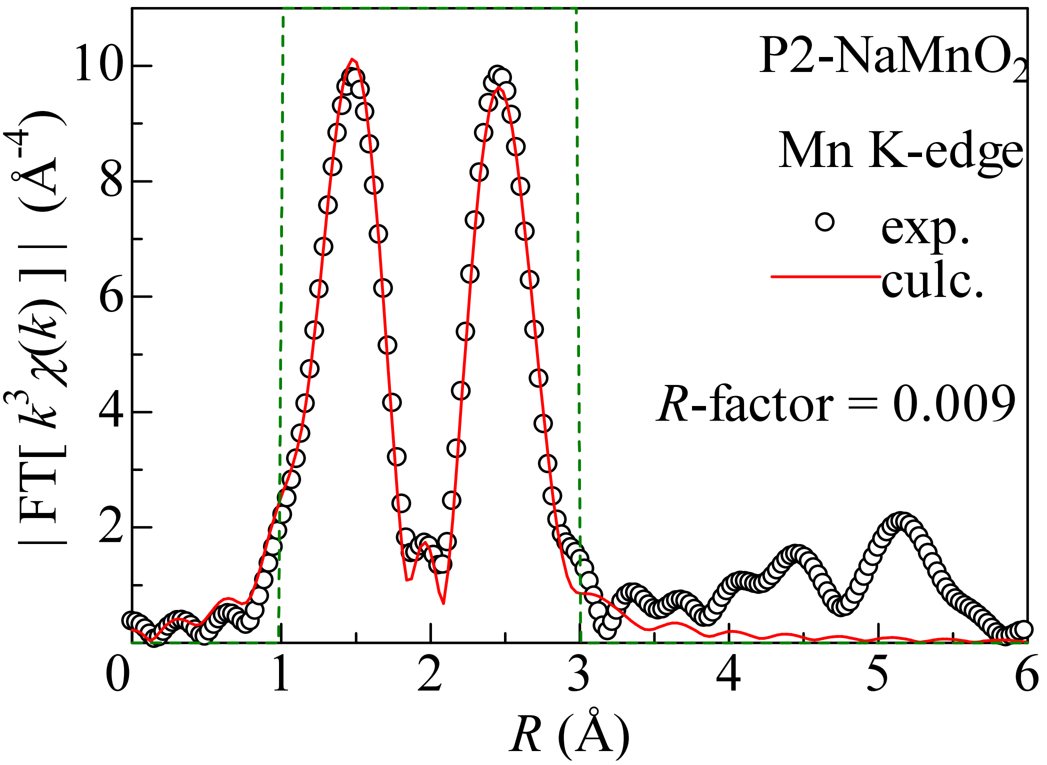

Fig. S9: FT[$\chi(k)k^3$]–R plot of Mn K-edge of P2-$NaMnO_2$. Red curve is the least-squares fitting with the EXFAS equation in the R range from 1Å to 3Å. The obtained parameters are the following.

Path	S_0^2	N_j	R (Å)	σ^2 (10^{-3} Å^2)	E_0 (eV)
Mn-O	0.73(8)	6	1.907(8)	5.9(12)	6543.1(13)
Mn-Mn	0.73	6	2.898(9)	7.0(10)	6543.1
Mn-O2	0.73	6	3.462(69)	13.4(109)	6543.1

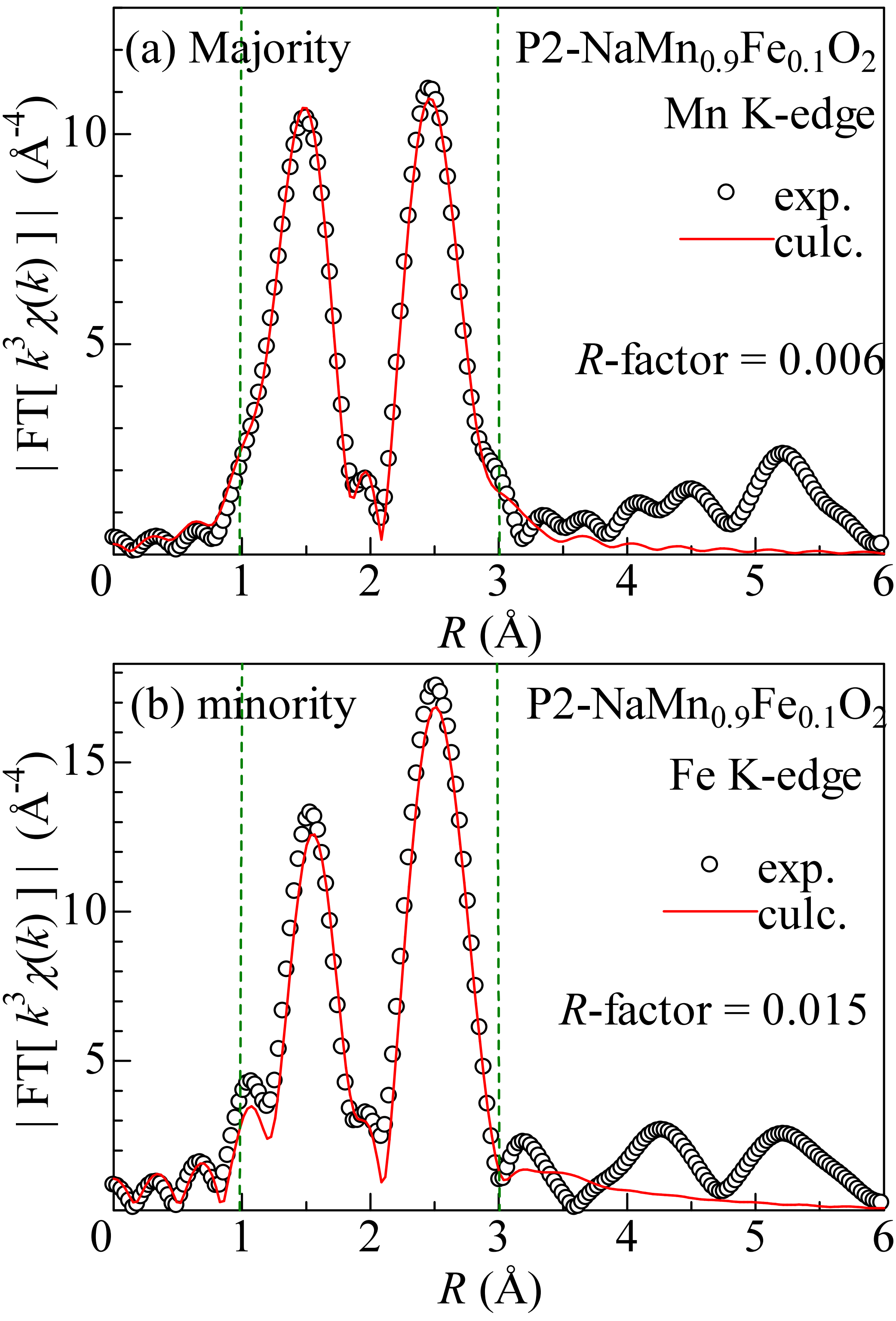

Fig. S10: FT[$\chi(k)k^3$]–R plots of (a) Mn K-edge and (b) Fe K-edge of P2-$NaMn_{0.9}Fe_{0.1}O_2$. Red curve is the least-squares fitting with the EXFAS equation in the R range from 1Å to 3Å. The obtained parameters are the followings.

majority Mn

Path	S_0^2	N_j	R (Å)	$\sigma^2 (10^{-3}$ Å$^2)$	E_0 (eV)
Mn-O	0.73	6	1.908(6)	5.4(5)	6543.6(9)
Mn-TM	0.73	6	2.901(6)	6.0(4)	6543.6
Mn-O2	0.73	6	3.485(43)	11.1(70)	6543.6

minority Fe

Path	S_0^2	N_j	R (Å)	$\sigma^2 (10^{-3}$ Å$^2)$	E_0 (eV)
Fe-O	0.69	6	1.998(12)	1.7(8)	7116.9(22)
Fe-TM	0.69	6	2.905(11)	2.1(6)	7116.9
Fe-O2	0.69	6	3.317(61)	3.0	7116.9

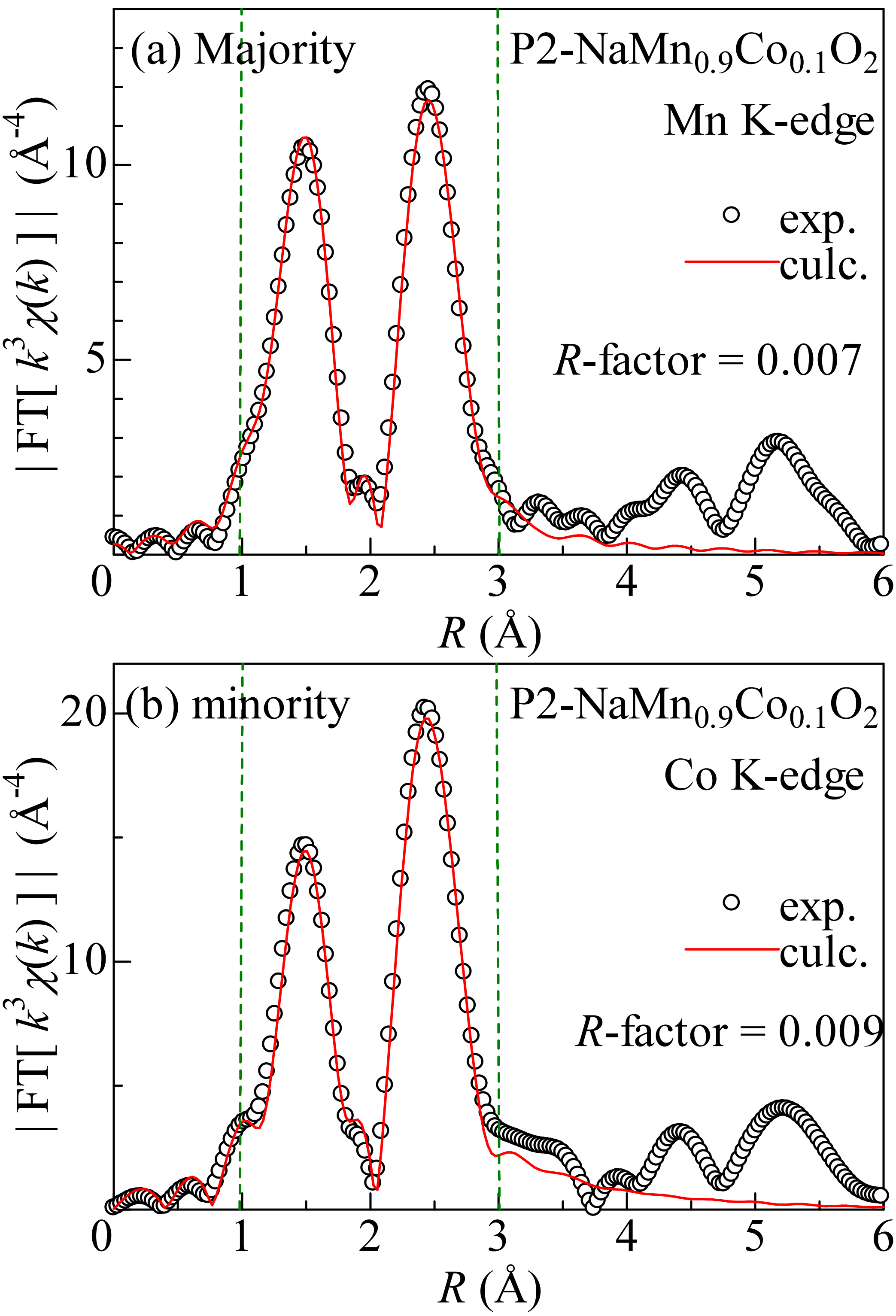

Fig. S11: FT[$\chi(k)k^3$]–R plots of (a) Mn K-edge and (b) Co K-edge of P2-$NaMn_{0.9}Co_{0.1}O_2$. Red curve is the least-squares fitting with the EXFAS equation in the R range from 1Å to 3Å. The obtained parameters are the followings.

majority Mn

Path	S_0^2	N_j	R (Å)	$\sigma^2 (10^{-3}$ Å$^2)$	E_0 (eV)
Mn-O	0.73	6	1.909(7)	5.4(6)	6543.7(10)
Mn-TM	0.73	6	2.880(7)	5.6(4)	6543.7
Mn-O2	0.73	6	3.453(50)	12.0(82)	6543.7

minority Co

Path	S_0^2	N_j	R (Å)	$\sigma^2 (10^{-3}$ Å$^2)$	E_0 (eV)
Fe-O	0.77	6	1.914(9)	2.6(7)	7713.1(17)
Fe-TM	0.77	6	2.848(8)	2.1(4)	7113.1
Fe-O2	0.77	6	3.296(77)	9.1(83)	7113.1

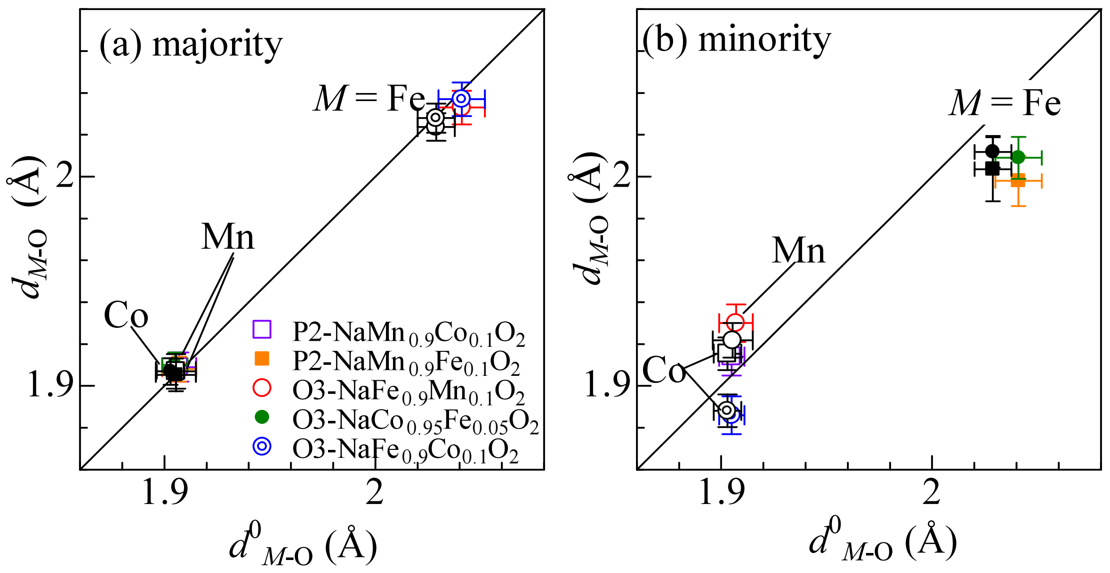

Fig. S12 Interatomic distances ($d_{M\text{-O}}$) to the nearest neighbor atom in the mixed layered oxides around (a) majority and (b) minority *M*s. Horizontal axes are the *M*-O distances ($d^0_{M\text{-O}}$) in the corresponding pure layered oxides, *i.e.*, O3-$NaFeO_2$, O3-$NaCoO_2$, and P2-$NaMnO_2$. Back marks represent the corresponding data obtained with including the contributions from the nearest neighboring O and *M* in the EXAFS analyses.

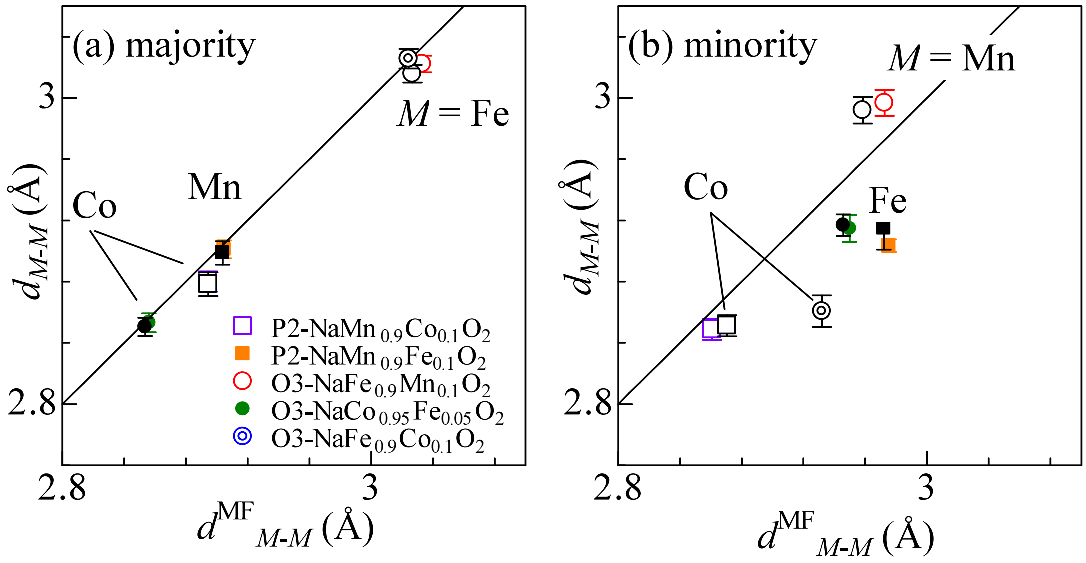

Fig. S13: Interatomic distances ($d_{M\text{-}M}$) of the next nearest neighbor TM in the mixed layered oxides around (a) majority and (b) minority. Horizontal axes are the mean-field values ($d^{MF}_{M\text{-}M}$) evaluated from the values of the pure layered oxides, *i.e.*, O3-$NaFeO_2$, O3-$NaCoO_2$, and P2-$NaMnO_2$. Back marks represent the corresponding data obtained with including the contributions from the nearest neighboring O and *M* in the EXAFS analyses.

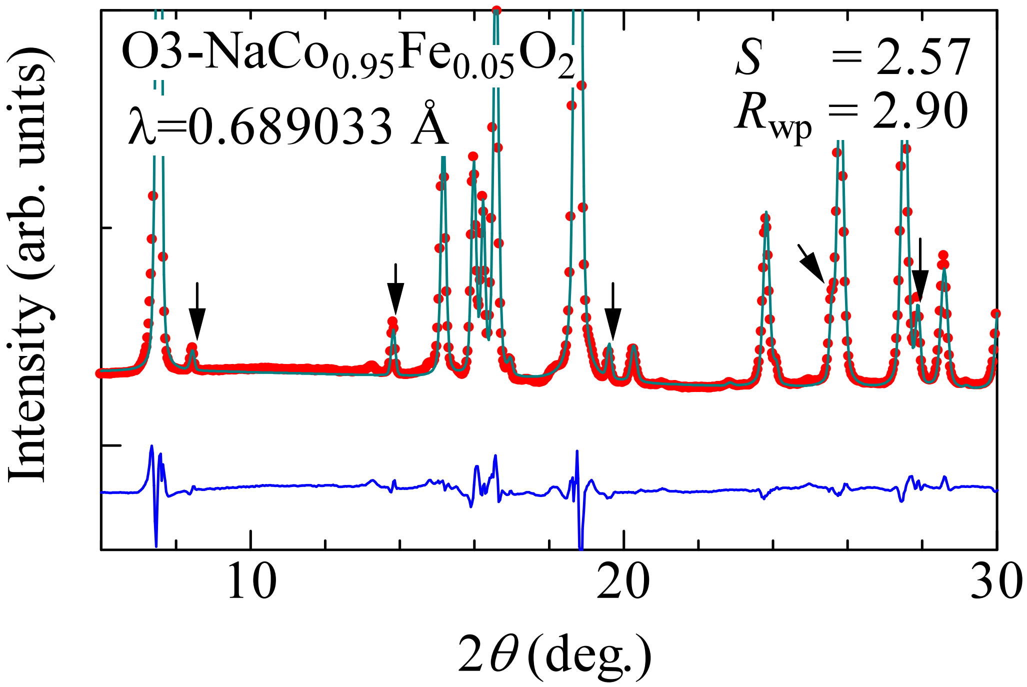

Fig. S14: Synchrotron-radiation X-ray powder diffraction pattern of O3-$NaCo_{0.95}Fe_{0.05}O_2$. The green curve is result of two-phase Rietveld refinement with the O3 ($R\bar{3}m$; Z= 3, hexagonal setting) and spinel (*Fd-3m*; Z = 8) phases. The blue curve is the difference between experimental data and calculation. The structural parameters of the O3 phase are fixed at the values in Table S5. The additional reflections (downward arrows) are well reproduced by the 5wt% spinel phase with a = 8.0805(2) Å.
